# Evolution of Human Scar-Related Ventricular Tachycardia Mapping for Exploring Mechanisms of Reentry Circuits

**DOI:** 10.31083/RCM43980

**Published:** 2025-11-17

**Authors:** Takuro Nishimura, Roderick Tung

**Affiliations:** ^1^Department of Cardiovascular Medicine, Institute of Science Tokyo, 113-8519 Tokyo, Japan; ^2^Banner-University Medical Center, The University of Arizona College of Medicine, Phoenix, AZ 85006, USA

**Keywords:** ventricular arrhythmia, mapping, catheter ablation, functional substrate

## Abstract

Ventricular tachycardia (VT) can originate from diseased myocardium resulting from ischemic or nonischemic cardiomyopathy. Scar-related VT is predominantly sustained by reentrant circuits within areas of myocardial scar. The therapeutic target within these circuits is the isthmus—an electrically insulated pathway bounded by electrical barriers. To elucidate the mechanisms of isthmus formation and the structural characteristics of VT circuits, electrophysiological mapping during VT has advanced in parallel with technological innovations, including intraoperative mapping, electroanatomical mapping, and, more recently, high-density mapping using multipolar catheters. We have recently characterized VT circuits involving the intramural component and proposed a hyperboloid model to conceptualize three-dimensional VT propagation. Furthermore, we demonstrated that the majority of isthmus boundaries are formed by anatomically fixed lines of conduction block, as identified by substrate mapping. Novel technologies, such as a frequency analysis of intracardiac electrograms and micro-mapping catheters for the coronary vessels, have also been developed to investigate intramural VT circuits.

## 1. Introduction

**Fig. 1.**
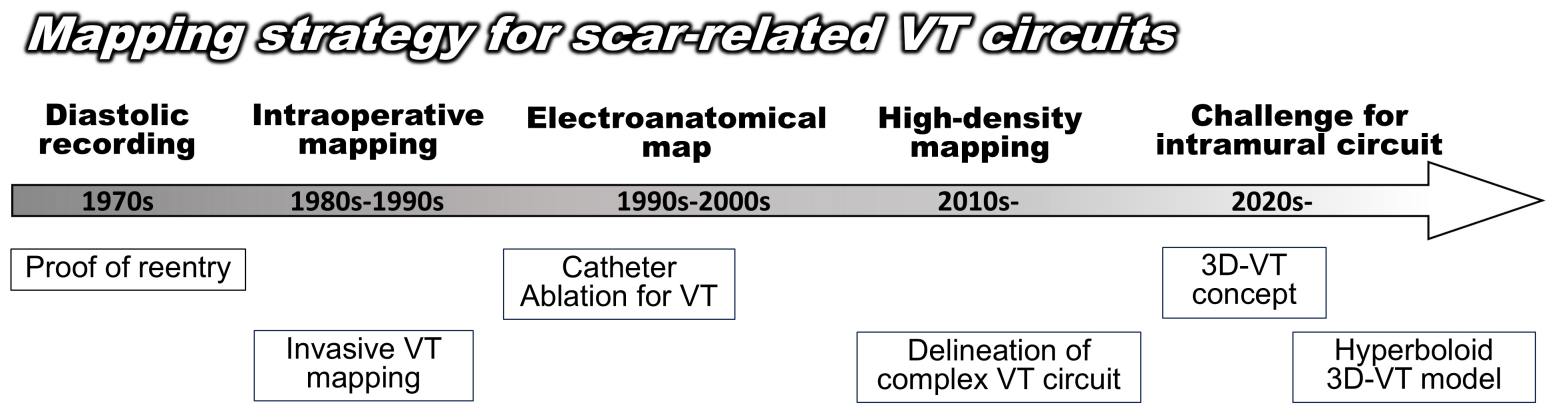
**Development of a mapping strategy for scar-related VT circuits**. 3D, 3 Dimensions; VT, ventricular tachycardia.

Ventricular tachycardia (VT) related to structural heart disease is a life-threatening arrhythmia and a major cause of sudden cardiac death. Most scar-related VT is based on a reentrant mechanism involving myocardial scar, resulting from ischemic cardiomyopathy (ICM) and nonischemic cardiomyopathy (NICM). Over the past half-century, various mapping techniques have been explored to elucidate the mechanisms underlying scar-related VT circuits (Fig. 1). The ideal target for treating reentrant VT is the critical isthmus, which can be identified by mapping during VT. However, in many cases, it is challenging because of hemodynamic instability during VT, preventing prolonged activation mapping. Substrate mapping strategies are required to assess the arrhythmogenic substrate constructing the VT circuits during the baseline rhythm [1].

This review describes the evolution of VT mapping techniques and the mechanisms that have been elucidated. By reviewing both the historical advances and recent data, it aims to provide a comprehensive perspective on the current status of scar-related VT mapping.

## 2. History on Mapping of Reentrant VT Circuits

In the 1970s, the mechanism of monomorphic VT following a myocardial infarction was believed to be reentry, based on a patterned induction and termination by pacing [2, 3, 4, 5]. In 1978, Josephson *et al*. [6] first reported continuous diastolic activity in humans, providing direct electrophysiological evidence of a reentrant mechanism. They used a single bipolar electrode placed within the aneurysm of patients with a post-myocardial infarction and recorded continuous diastolic potentials. In 1989, Waldo and Henthorn [7] reported the method of interpreting the circuit characteristics based on the response to overdrive pacing performed during tachycardia as transient entrainment. In the 1990s, entrainment mapping was established by Stevenson *et al*. [8] and Ellison *et al*. [9] as the gold standard for electrophysiologically identifying the components of the circuit.

**Fig. 2.**
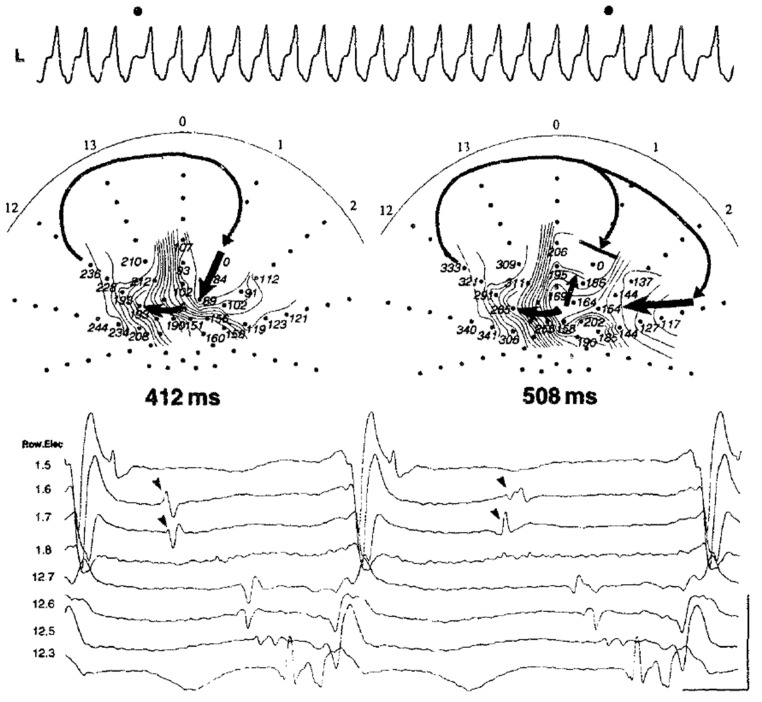
**Ventricular tachycardia circuit delineation during a change in the QRS morphology with a balloon array catheter during intraoperative mapping**. Changes in the entrance and shared isthmus are associated with variations in the tachycardia cycle length, as reported by Downar *et al*. [17]. This figure is reproduced from the original manuscript with permission from Elsevier.

The delineation of myocardial activation during sustained VT using a multielectrode catheter was introduced to understand the actual dimensions, structure, and mechanism of the reentrant VT circuit. Activation mapping was initially developed in the 1970s and 1980s through intraoperative mapping during the surgical treatment of VT in patients with post-myocardial infarction [10, 11, 12, 13]. Miller *et al*. [13] reported activation maps of 55 patients to guide subendocardial resections in 1985. They demonstrated that 90% of VTs originated from a focal area within a 6 cm^2^ region, while the remaining 10% involved a circuit rotating around the aneurysm. Based on these findings, a mapping-guided surgical subendocardial resection for eliminating the entire tachycardia circuit was proposed [13]. In some intraoperative analyses, electrode-covered sock arrays placed on the epicardial surface and endocardial balloon electrode arrays were utilized to map the ventricular tachycardia activation [14, 15, 16]. Notably, these mapping techniques provided higher accuracy compared to contact mapping using a high resolution multielectrode catheter, which is currently used, as a single reentrant cycle activation could be delineated due to the fixed position of the electrodes. In 1988, Downar *et al*. [14] reported the successful delineation of VT activation using a balloon array with 112 electrodes in the left ventricle. With the patient on cardiopulmonary bypass and at normothermia, the deflated balloon array was passed through a left atriotomy, across the mitral valve, and into the left ventricle. By recording the propagation of premature stimuli and tracking the initiation of reentry beat by beat, the isochronal map demonstrated that reentrant activation was triggered by a conduction delay and the formation of a functional arc of block [14]. In addition, they reported that the pleomorphism of sustained VT was due to a wavefront alternation. When one of the two entry pathways intermittently became blocked, the cycle length varied intermittently, but the QRS morphology remained unchanged. In contrast, when a block occurred in one of the exit pathways, the activation shifted to an alternative exit, leading to significant changes in both the ventricular activation and QRS morphology [17] (Fig. 2).

**Fig. 3.**
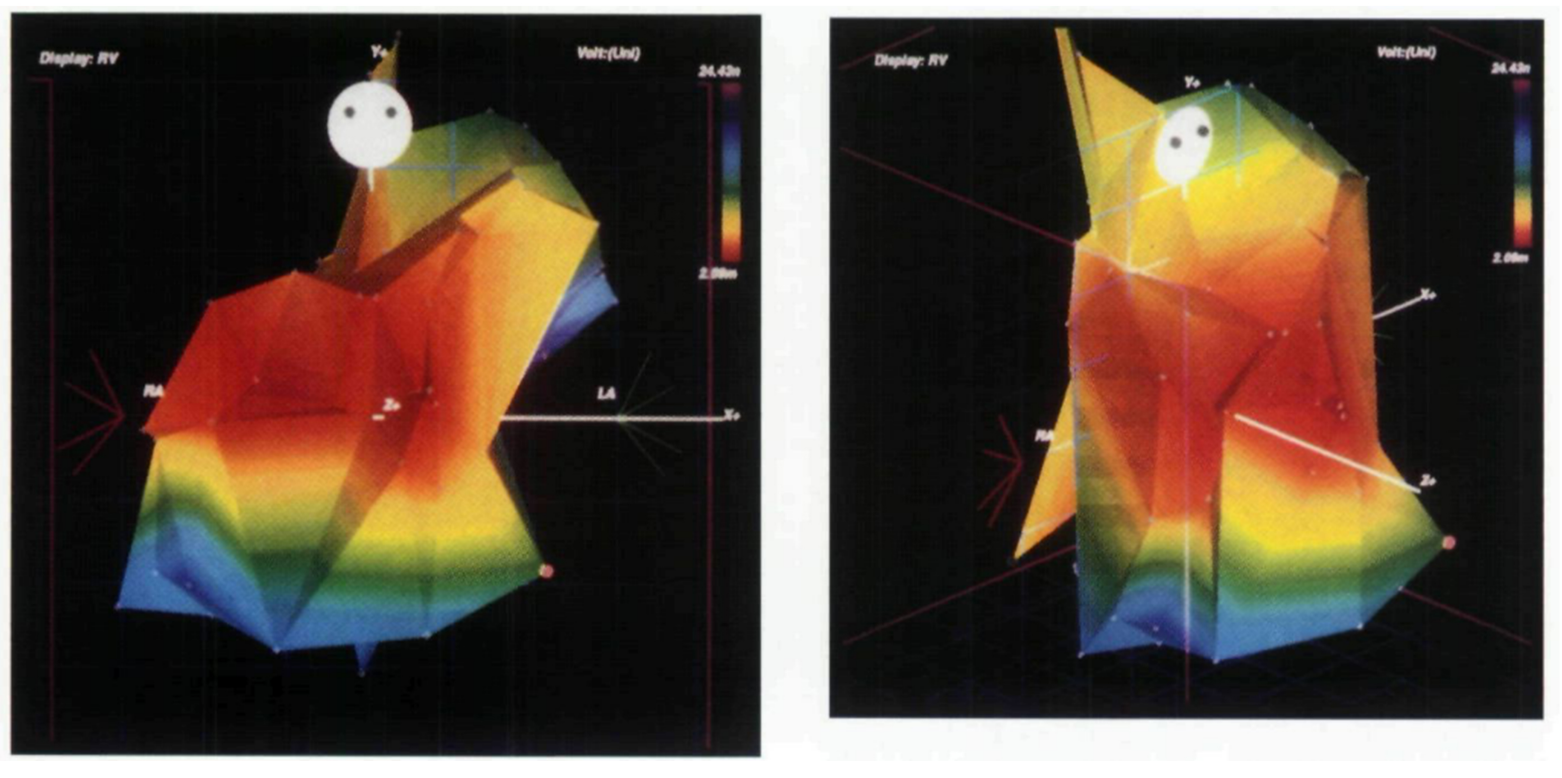
**Voltage map in right ventricle created by point-by-point approach**. Mapping of the right ventricle in a postoperative patient with tetralogy of Fallot. This is an early report of electroanatomical mapping, created using a point-by-point approach with a single bipolar electrode. The red area indicates a relatively low-voltage region extending from the anterior wall to the free wall of the right ventricle. This figure is reproduced from the original manuscript with permission from John Wiley and Sons [19].

In the late 1990s, electroanatomical mapping incorporating the three-dimensional geometry was introduced for the field of cardiac electrophysiology (Fig. 3) [18, 19]. Around the same time, radiofrequency catheter ablation also began to be applied to VT [20]. We have become able to assess the catheter position and electrogram data less invasively. The ventricular geometry was constructed in a point-by-point fashion using an ablation catheter with tens to hundreds of mapping points. In 2002, de Chillou *et al*. [21] reported the complete maps of 33 reentrant hemodynamically stable VT activations with 144 ± 69 points using a CARTO system (Biosense Webster, Diamond Bar, CA, USA) in patients post-myocardial infarction. They successfully delineated both single-loop and double-loop circuits, demonstrating that the isthmus is shared among multiple circuits. In this report, the reentrant isthmus of ischemic VT was measured to be approximately 31 mm in length and 16 mm in width. Soejima *et al*. [22] reported that the reentrant VT isthmus was formed between electrically unexcitable scar (EUS) caused by an infarction, indicating that an insulator was forming the VT isthmus. Zeppenfeld *et al*. [23] later expanded on Soejima’s findings of VT after the repair of congenital heart disease.

Advances in epicardial mapping have further enhanced VT mapping. Although epicardial mapping had been performed using a surgical approach, Sosa *et al*. [24] reported a method in which a catheter was inserted into the epicardium via a parasternal puncture for mapping and VT ablation in 1996. Since then, the efficacy and safety of epicardial mapping and ablation of VT have been reported in many institutions [25, 26, 27, 28].

## 3. VT Circuit Analysis Using High-Density Mapping

**Fig. 4.**
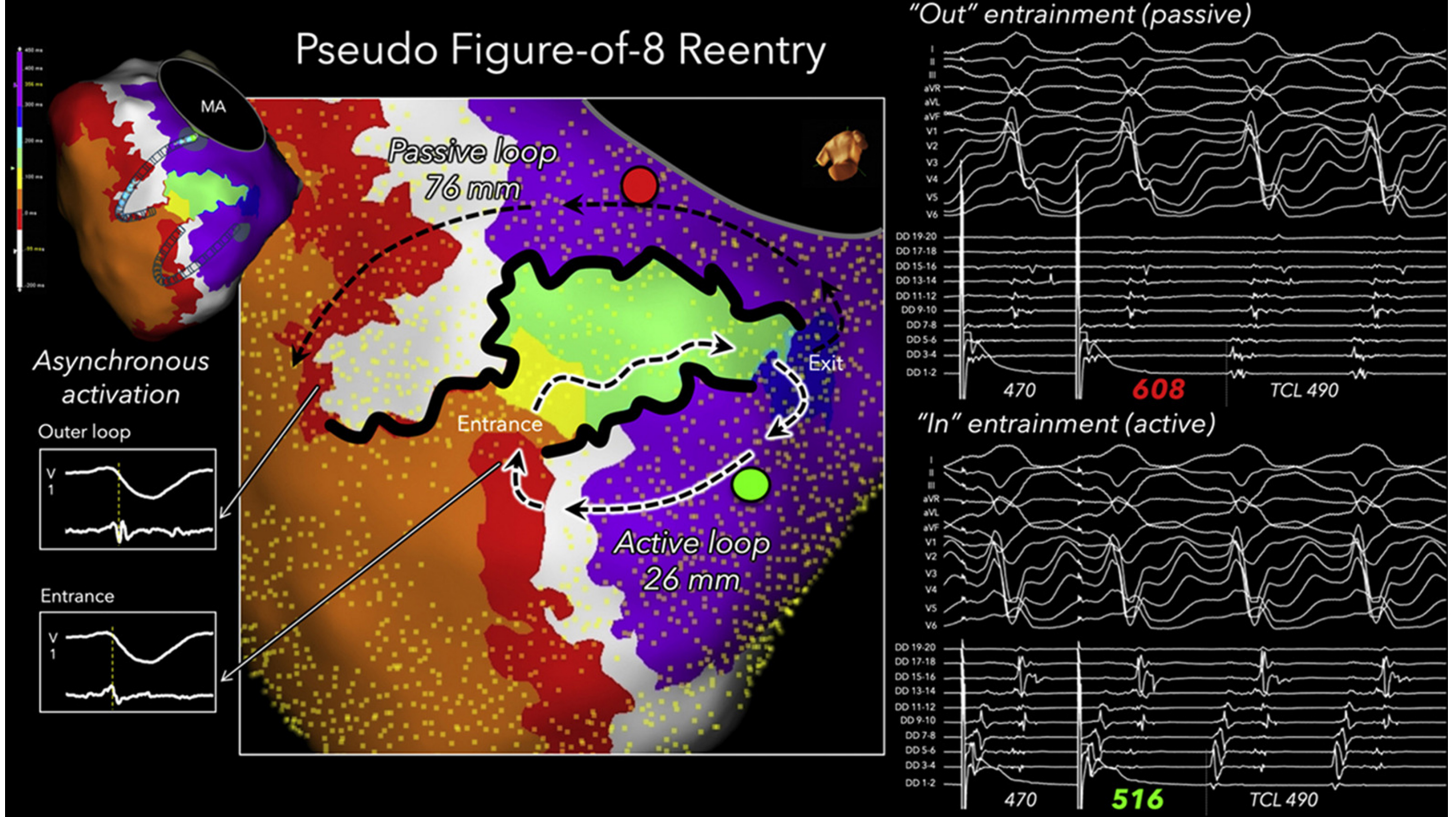
**Figure-of-eight shaped VT circuit with single loop mechanism**. A figure-of-eight–shaped VT circuit was delineated in the left ventricular endocardium of a patient with ischemic cardiomyopathy. The activation loop with a long isthmus boundary, indicated by the non-highlighted black dashed arrows, was identified as a passive bystander loop based on entrainment mapping performed at the red point. In contrast, the active reentrant loop was highlighted in white, which was confirmed by entrainment mapping at the green point. In this passive loop, the red isochrone corresponded to outer loop activation, whereas in the active circuit, the same isochrone represented entrance activation. This figure is reproduced from the original manuscript with permission from Elsevier [36]. VT, ventricular tachycardia; MA, mitral annulus; DD, duodecapolar linear catheter; F8, double loop figure-of-eight; PPI, post pacing interval; TCL, tachycardia cycle length.

Since the late 2000s, multipolar catheters specialized for mapping have been introduced [29, 30]. Unlike the conventional point-by-point mapping method, these catheters enable the creation of high-density maps with thousands of points. The circuit of hemodynamically unstable VTs became possible to be delineated in a short time [31, 32, 33]. Furthermore, this advancement allows for a detailed assessment of the complex structure of the circuit, its precise size, and the conduction velocity of the VT activation [29, 33, 34]. In 2016, Anter *et al*. [34] analyzed 21 VT circuits using high-density mapping in a postinfarction swine model. They utilized the Orion 64-electrode minibasket catheter (0.4 mm^2^ electrode, 2.5 mm center-to-center spacing, Boston Scientific, Cambridge, MA, USA) and acquired 8240 ± 3326 points over a median duration of 8 minutes during VT. Their analysis highlighted the limitations of entrainment mapping accuracy, showing that the isthmus defined by traditional entrainment criteria exceeded the dimensions identified by high-density mapping by 32 ± 18%. Furthermore, they demonstrated that the conduction velocity (CV) decreased as the wavefront curved at the entrance and exit compared to within the isthmus, as previously reported in experimental canine models [35]. In a human analysis using the Orion catheter, Martin *et al*. [29] investigated 36 scar-related VTs in 2018. We analyzed figure-of-eight shaped VT circuits with Ensite system (Abbott, Abbott Park, IL, USA) with a multielectrodecatheter (HD Grid Advisor: 1 mm ring, 3 mm edge-to-edge spacing and Livewire: 1 mm ring, 2 mm edge-to-edge spacing, Abbott). Most figure-of-eight shaped VT circuits had asymmetrical isthmus with different length of isthmus boundaries. Asymmetric entrainment responses in the outer loops also suggested that a true figure-of-eight circuit is rare. A single dominant active loop appears to be the key mechanism sustaining scar-related reentrant VT [36] (Fig. 4).

**Fig. 5.**
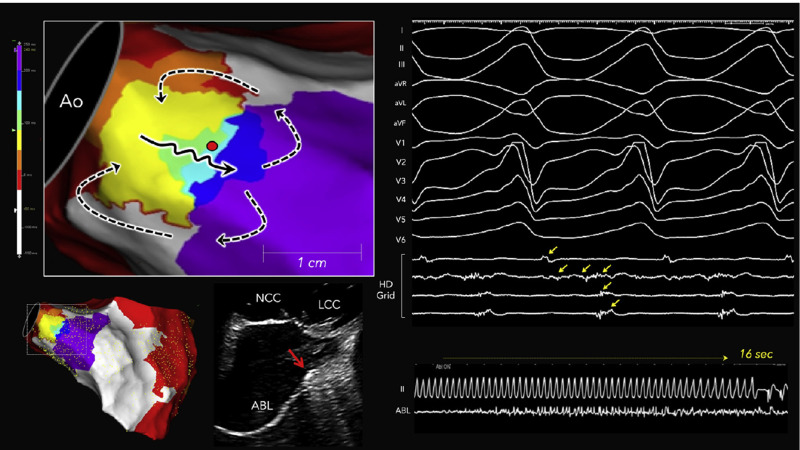
**Localized reentry circuit within the periaortic septum in a nonischemic cardiomyopathy patient**. The yellow arrows indicate diastolic potentials. Long fractionated potentials spanning the entire diastolic phase were recorded by a single bipolar electrode located in the periaortic septum during VT. The black solid and black dashed arrows indicate the isthmus and outer loop activation in the reentrant circuit, respectively. The red dot indicates the site where radiofrequency ablation successfully terminated the tachycardia, and the intracardiac echocardiographic image shows the ablation catheter positioned at the periaortic septum (red arrow). This figure is reproduced from the original manuscript with permission from Elsevier [38]. ABL, ablation catheter; Ao, aorta; ICE, intracardiac echocardiography; LCC, left coronary cusp; NCC, noncoronary cusp.

Several reports have measured the size of the isthmus in human VT using high-density mapping. Tung *et al*. [37] showed that 28% of circuits had a central isthmus with a minimal dimension of <1 cm, and 55% had a minimal dimension of <1.5 cm. This is nearly equivalent to the size reported by intraoperative mapping [13, 21]. We have proposed a definition for localized reentry where the minimal dimension of the isthmus is less than 1.5 cm. In such cases, the entirety of diastole can be recorded within 1–4 bipole pairs, signifying rotation around a small region [38, 39] (Fig. 5). Focusing on the diastolic potentials recorded within the isthmus during VT, the duration of the longest diastolic electrogram was inversely correlated with the dimensions of the isthmus and predictive of rapid VT termination by a single radiofrequency application [33]. Additionally, it was shown that diastolic potentials during VT have significantly higher amplitudes compared to electrograms recorded at the same site during sinus rhythm [29].

**Fig. 6.**
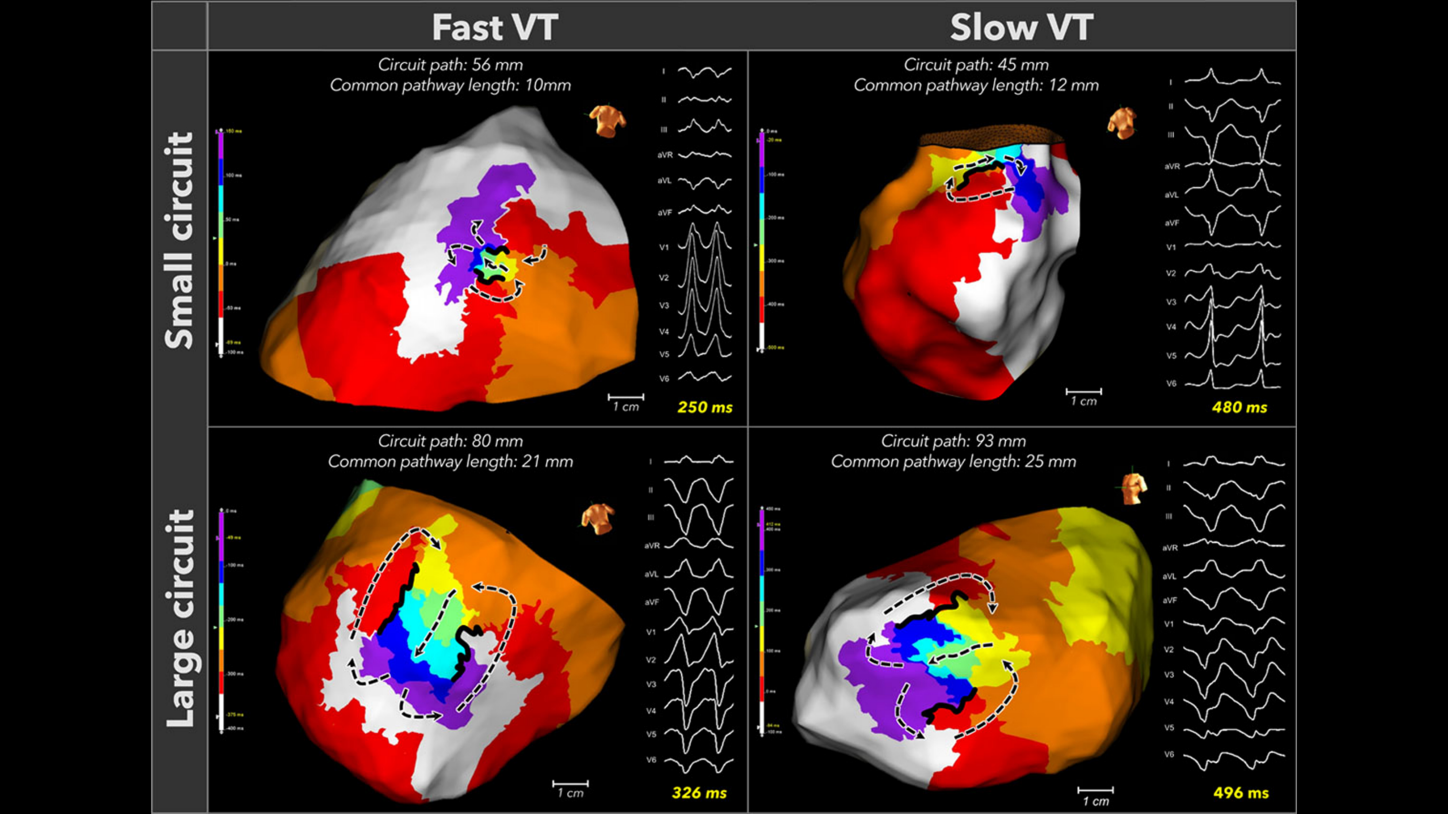
**Fast and slow VTs exhibit both small and large reentrant circuits**. Four representative circuits that demonstrated the circuit size do not determine the tachycardia cycle length. This figure is reproduced from the original manuscript with permission from Wolters Kluwer Health, Inc [33].

The analysis of the cycle length and circuit size in 54 human scar-related VTs revealed that the isthmus dimensions did not correlate with the VT cycle length in both ICM and NICM (Fig. 6, Ref. [33]). Instead, the primary factor determining the VT cycle length was the conduction velocity of the outer loop [33]. These data provide novel insights, suggesting that the outer loop may influence the characteristics of VT across the entire spectrum of myocardial substrates, ranging from normal tissue to dense scar.

## 4. 3D Structural Insights Into Scar-Related VT Circuits

**Fig. 7.**
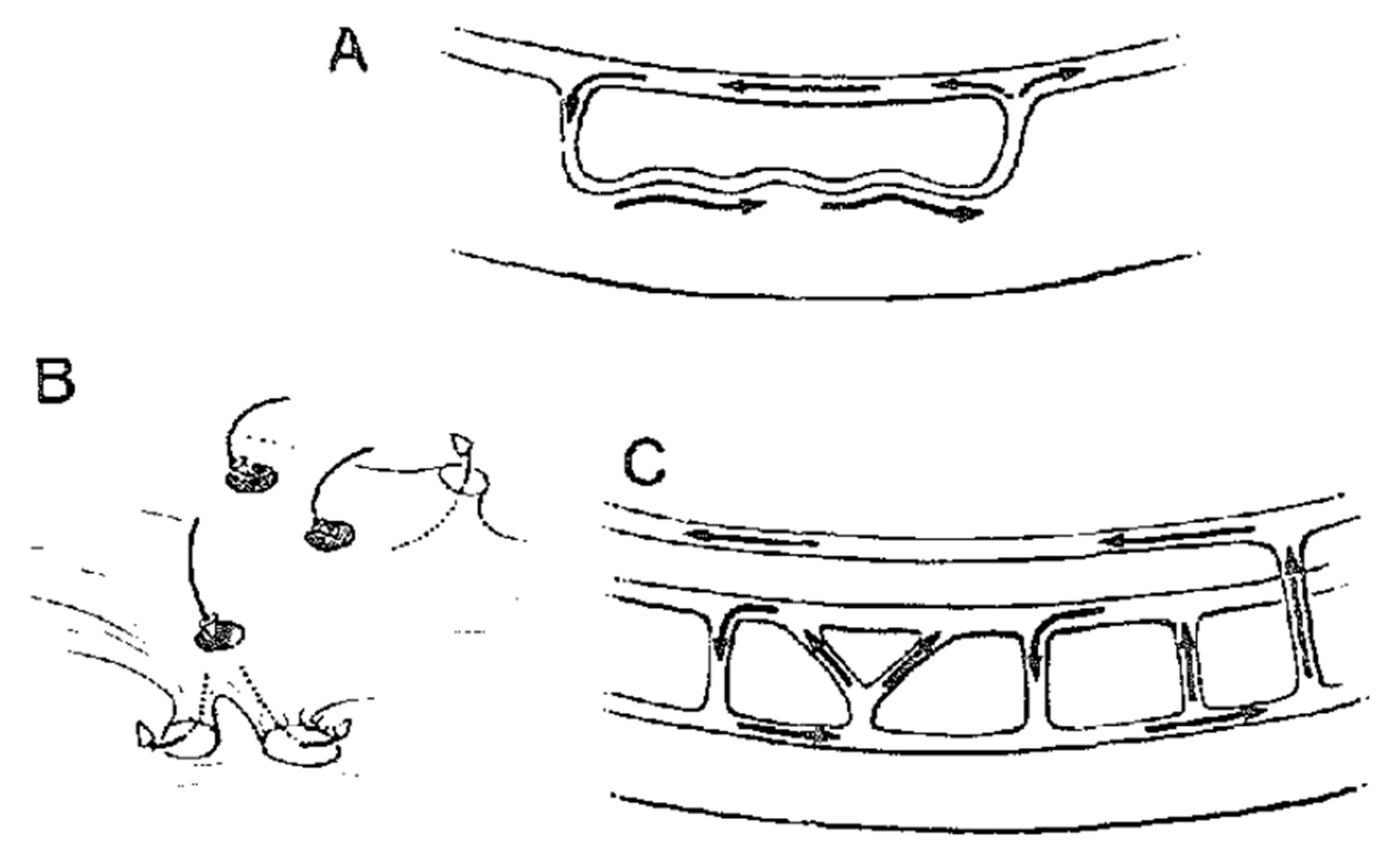
**Concept of intramural activation during scar-related reentry**. Downar *et al*. [16] proposed that intramural surviving myocardium was an essential part of the VT circuit. This figure is reproduced from the original manuscript with permission from Elsevier. (A) Schematic illustration of a single-path reentry incorporating the intramural component. (B) Schematic illustration of a reentry circuit with multiple connections, potentially acting like a “sinkhole” on the cardiac surface. (C) Schematic illustration depicting reentry established through involvement of the mid-myocardium and the left bundle branch system.

Intraoperative mapping indicated that the circuit can include intramural components, meaning it is not confined to a single cardiac surface. In 1987, Harris *et al*. [40] performed intraoperative mapping on both the epicardium and endocardium during 45 VT episodes in patients with ICM. They reported that continuous activation throughout the VT cycle was infrequently observed [40]. de Bakker *et al*. [41] reported that the majority of endocardial activation during ischemic VT followed a centrifugal pattern and, based on historical findings, demonstrated that intramural (subendocardial) anisotropy can support a reentrant circuit. They suggested that the circuit structure included intramural components, but noted that the spatial resolution of the balloon electrode (with an interelectrode distance of about 1.2 cm) was insufficient for a detailed analysis of the activation near the site of origin. Downar *et al*. [16] also proposed that intramural surviving myocardium was an essential part of the VT circuit based on high-density balloon mapping of the endocardium (Fig. 7, Ref. [16]). After those reports, Pogwizd *et al*. [42] reported direct intramural activation mapping during VT in patients with ICM in 1992. They used multiple plunge needles during cardiac surgery and demonstrated a three-dimensional circuit structure extending across the endocardium, myocardium, and epicardium in human VT [42]. Recently, Bhaskaran *et al*. [43] reported plunge needle mapping in both ICM and NICM patients and validated the intramural activation during VT.

**Fig. 8.**
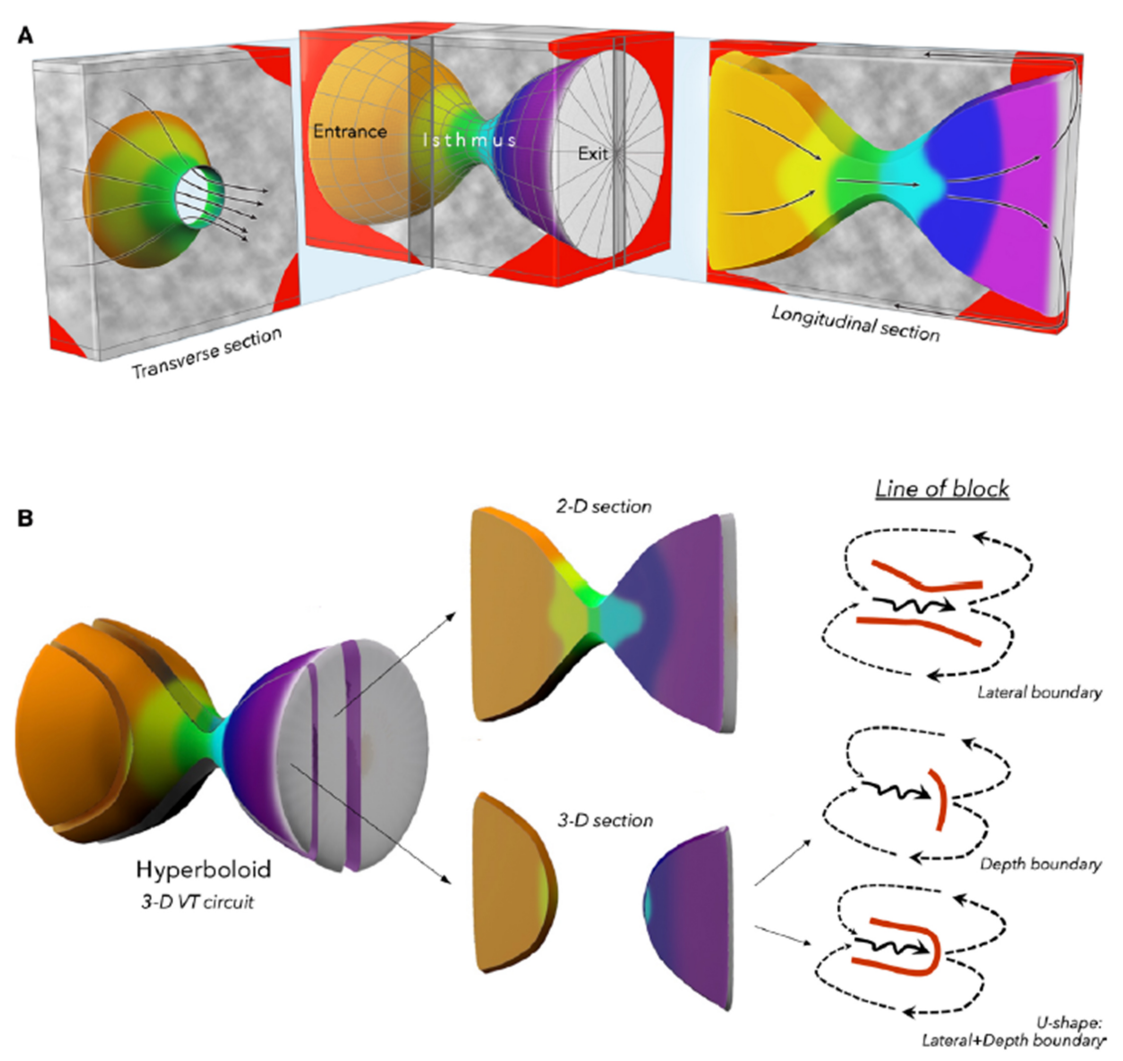
**A hyperboloid model for the 3D structure of a ventricular tachycardia circuit**. (A) We proposed that the 3D VT circuit can be illustrated by a conic section through a hyperboloid structure. Gray regions indicate fibrosis, which may serve as depth boundaries that confine the intramural isthmus activation. (B) A longitudinal conic section that cuts through the isthmus would portray the 2D VT circuit. A longitudinal conic section that does not cut through the isthmus would show an activation gap. A line of block during baseline rhythm can serve as both a lateral boundary in one plane and a depth boundary with activation below the plane in 3-D circuits. This figure is reproduced from the original manuscript with permission from Wolters Kluwer Health, Inc [44]. 2D, 2 Dimensions.

In 2020, Tung *et al*. [37] presented a complex three-dimensional (3D) VT circuit structure using high-density mapping. Simultaneous endocardial and epicardial mapping (SEEM) was performed for 83 circuits (ICM: n = 44, NICM: n = 39). Those results suggested that most circuits have a 3D structure, including transmural or intramural components, while only 17% of circuits are activated in a 2D plane restricted to a single myocardial surface. The occurrence of a 3D circuitry was more frequent in ICM compared with NICM (73% vs. 49%; *p* = 0.025). Another unique aspect of that report is that the exit of the isthmus is infrequently confined to a single myocardial surface, highlighting the limitations of predicting “epicardial VT” based on the QRS morphology. The most challenging circuit to identify is a completely intramural circuit, which manifests as passive focal activation on both surfaces, forming a “focal-focal” pattern. In such cases, the only viable approach of catheter ablation is to target the center of the focal activation, despite the possibility that the critical isthmus may be located several centimeters away; indeed, their analysis has shown that the distance between the mid-isthmus and the exit was 43 mm (range: 20–98 mm). Jiang *et al*. [39] analyzed 30 VT circuits with SEEM in patients with arrhythmogenic right ventricular cardiomyopathy. They showed that the extent of the disease progression from the epicardial side may determine the degree of transmural involvement of the circuit. Notably, in patients with a limited endocardial scar area and preserved right ventricular ejection fraction, localized reentrant circuits were predominantly observed on the epicardium [39]. In NICM, localized reentry circuits can be formed in the periaortic region. Our analysis showed that patients with 3D periaortic VT circuits had a higher recurrence rate of VT after catheter ablation compared to those without 3D circuits (73% vs. 37%; *p* = 0.028) [38]. We have recently proposed that a 3D VT circuit can be conceptualized as a cross-section of a hyperboloid model (Fig. 8, Ref. [44]). When this cross-section includes the middle-constricted portion, it can represent a 2D VT model. In contrast, in the case of a 3D VT model, the constricted portion of the hyperboloid represents intramural isthmus conduction, illustrating how the isthmus conduction is confined to the intramural space from the epicardium. This model defines a boundary that regulates the depth of the isthmus conduction from the cardiac surface (depth boundary) [44].

Several less invasive methods for predicting a 3D VT without relying on activation mapping have also been reported. Even in cases of unmappable VT, the possibility of identifying the 3D VT isthmus using pace-mapping from both the endocardium and epicardium has been demonstrated [45]. Toloubidokhti *et al*. [46] reported the noninvasive delineation of the 3D VT circuit activation in a porcine model using a 120-lead electrocardiogram and cardiac computed tomography (CT) imaging.

## 5. Mechanism of VT Isthmus Boundaries

Whether the isthmus boundary of a scar-related VT circuit is functionally defined or determined by a preexisting fixed line of block has been a subject of debate. In studies using animal models of myocardial infarctions, molecular-level abnormalities, such as changes in the refractoriness due to ion channel dysfunction and gap junction abnormalities, have been reported to be associated with the formation of VT isthmus boundaries [47, 48]. There are reports suggesting that the VT isthmus boundary is functionally formed, as evidenced by the absence of abnormal potentials at the VT isthmus site during sinus rhythm [49]. Ciaccio *et al*. [35] suggested that abrupt changes in the myocardial mass within the infarct border zone could induce a source-sink mismatch, potentially contributing to the formation of the isthmus boundary. Another finding suggesting a functionally isthmus boundary formation is the recording of a wavefront traversing the isthmus boundary during VT in an animal model [34, 50]. This recording suggests that the isthmus boundary was created by slow transverse conduction, rather than by conduction block.

On the other hand, Soejima *et al*. [22] reported a reentrant VT isthmus formed between areas of EUS caused by an infarction. EUS was defined as an uncaptured scar identified by pacing, indicating that an insulating barrier was forming the VT isthmus [22]. de Chillou *et al*. [21] reported that the isthmus boundary was formed by the mitral annulus, scar area, and a line of conduction block, characterized by split potentials with a width of ≥50 ms, around which the reentrant circuit revolved. Those reports suggest that the VT isthmus boundary is formed by a fixed conduction block, which is a key target for catheter ablation [51, 52].

## 6. Modification of the Functional Substrate in Scar-Related VT

The ideal target for catheter ablation is the isthmus identified by VT activation mapping. However, in unmappable VT cases, ablation is guided by predicting an isthmus formation from the substrate recorded during sinus rhythm. The strategy for substrate modification in scar-related VT has evolved from voltage map-guided substrate evaluation. Radiofrequency ablation targeting abnormal voltage areas can prevent VT induction and reduce recurrence [53, 54, 55, 56]. Since the late 2010s, substrate evaluation using activation mapping during sinus rhythm has been reported. Several reports have demonstrated that wavefront discontinuities delineated by high-density mapping spatially coincide with the isthmus of the ventricular tachycardia (VT) circuit [57, 58, 59]. Aziz *et al*. [60] reported that targeting the deceleration zone, which is located proximal to the late potentials rather than the delayed excitation caused by diseased myocardium, results in a more efficient VT treatment compared to voltage-guided scar homogenization.

Recently, there have been reports that activation maps created with multiple wavefronts can depict substrates that may be masked when using only a single wavefront analysis. The concept was based on the report by Jaïs *et al*. [61] in 2012 regarding local abnormal ventricular activities (LAVA), as defined. They demonstrated that these abnormal electrograms become evident depending on the direction of the myocardial activation. That finding suggests that identifying all substrates with only one wavefront is challenging. High-density mapping has further demonstrated that the substrate delineation can change dynamically depending on the wavefront propagation [57, 62].

**Fig. 9.**
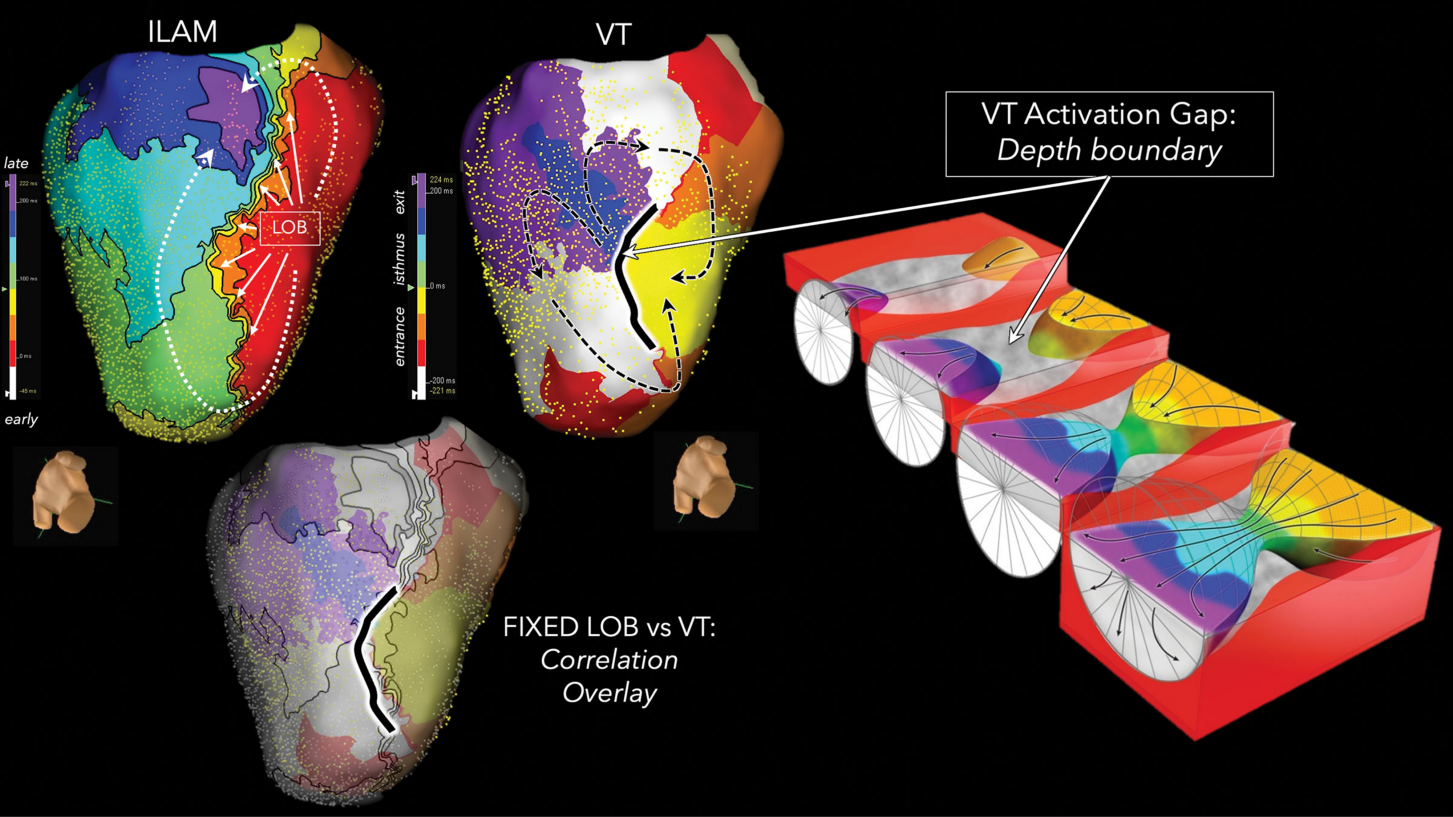
**Correlation between the line of conduction block during sinus rhythm and the VT circuit**. Isochronal late activation map during sinus rhythm exhibited an LOB on the inferior wall of the left ventricle. This LOB co-localized to a depth boundary during a 3D-VT, whereby a surface discontinuity was present during the intramural isthmus activation (green and cyan isochrones not recorded from the endocardium). The black dashed lines indicate VT activation. This figure is reproduced from the original manuscript with permission from Wolters Kluwer Health, Inc [44]. ILAM, isochronal late activation map; LOB, line of conduction block.

Theoretically, split potentials are created when a wavefront collides with a line of conduction block (LOB), rotates around its edge, and results in the recording of a second component. This phenomenon is most apparent when the wavefront direction is perpendicular to the LOB. Conversely, when the wavefront propagates parallel to the LOB or arrives simultaneously from both directions, the local electrogram is not split, and the LOB may become concealed. We proposed “differential pacing” within or near a deceleration zone to unmask anatomically fixed LOBs [44]. The substrate maps from 106 patients with scar-related VT (ICM: 58%, NICM: 42%) were analyzed. In 92% of deceleration zones where differential pacing was applied, LOBs with a width exceeding 20 ms were identified. Furthermore, the detected LOBs were largely spatially consistent with the isthmus boundaries (69% of 2D lateral boundary and 79% of 3D depth boundaries), suggesting that anatomically fixed LOBs form the isthmus boundaries (Fig. 9, Ref. [44]). In 14% of VT circuits, an extension of the LOB forming the isthmus boundary during VT was observed. This phenomenon is likely due to the rate-dependent functional extension of the block from the edges of the original LOB. As a substrate assessment strategy, identifying both anatomically fixed and rate-dependent LOBs may allow for a more precise delineation of the substrate involved in the isthmus formation. Beyond the delineation of LOB as boundaries of the isthmus, it is also essential to identify vulnerable regions prone to conduction slowing and unidirectional block, which are critical prerequisites for re-entry. VT initiation requires a critically timed extra stimulus with appropriate wavefront directionality, forming the basis of functional extra stimulus dynamic substrate mapping [63, 64]. This approach highlights the dynamic properties of the substrate and complements structural mapping for a more balanced strategy in VT ablation. A contemporary meta-analysis directly compared extra stimulus mapping with static functional mapping (performed during intrinsic spontaneous rhythm or under continuous ventricular pacing). This study demonstrated that extra stimulus mapping was independently associated with a lower VT recurrence rate [65]. Incorporating these findings underscores the clinical impact of novel mapping strategies, providing a more comprehensive perspective that integrates both mechanistic rationale and outcome-based evidence.

Traditionally, the distinction between near-field and far-field electrograms has been qualitatively described using terms such as “sharp” or “dull”. With the introduction of the novel Omnipolar Technology (OT) Near Field Software (Abbott, Abbott Park, IL, USA), it is possible to quantitatively analyze the frequency characteristics of local electrograms, enabling a more objective assessment. Mayer *et al*. [66] demonstrated that peak frequency analysis with a threshold of ≥200 Hz could highlight low-voltage regions corresponding to the VT isthmus, thus enabling identification of its critical components. While this approach provides additional mechanistic insights, its diagnostic performance may be lower compared with functional substrate mapping strategies, and further validation is warranted.

## 7. Mapping Intramural Components of the VT Circuit

High-density mapping has revealed complex circuit structures; however, intramural activation in three-dimensional ventricular tachycardias (3D-VTs) remains largely unknown. Mapping the wavefront penetration into the myocardial layers is challenging and the intramural substrate in NICM is particularly difficult to modify with radiofrequency ablation [67, 68]. Shirai *et al*. [69] reported the difference in entrainment mapping between ICM and NICM. The isthmus of NICM VTs is less identifiable even with both endocardial and epicardial entrainment mapping. Delayed enhancement on magnetic resonance imaging (MRI) or CT allows for the assessment of the extent of nonischemic potential arrhythmogenic substrates [70, 71]. We analyzed the MRI data from 25 NICM patients and demonstrated that the extent of late gadolinium enhancement in the ventricular septum correlates with the number of inducible VTs and the success rate of catheter ablation [72].

**Fig. 10.**
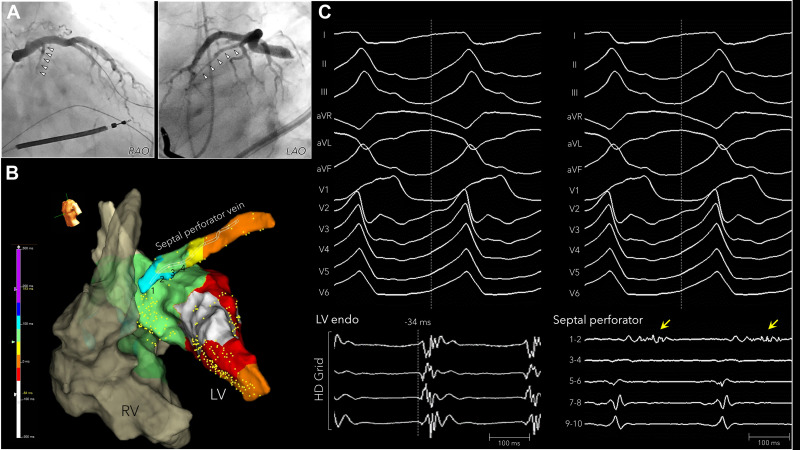
**Intramural mapping within the coronary venous septal branch with an over-the-wire microelectrode catheter**. A nonischemic cardiomyopathy patient with a septal substrate. (A) Coronary venography showed a large septal perforator vein. (B) The earliest activation on the left ventricular septum was –32 ms from the QRS onset of the VT. (C) A long, fractionated diastolic potential (yellow arrow) was recorded from electrodes 1–2 of the over-the-wire microelectrode catheter within the septal perforator vein. This figure is reproduced from the original manuscript with permission from Elsevier [74]. LV, left ventricle; RV, right ventricle; RAO, right anterior oblique; LAO, left anterior oblique.

One possible approach to map intramural activation is mapping the septal branches of the coronary vessels. Briceño *et al*. [73] reported intramural mapping of the NICM septum using unipolar recording from a wire. The usefulness of microelectrode catheters for bipolar recordings to map 3D-VTs has also been reported [74] (Fig. 10). We successfully mapped the septum in 10 cases using an over-the-wire catheter (EPstar Fix AIV [2.7 Fr]; Japan Lifeline, Tokyo, Japan), not only through the coronary veins but also via branches of the coronary arteries, and demonstrated its safety and clinical utility [75]. Another potential approach involves the use of a needle catheter to directly penetrate the myocardium and record electrical signals [42, 43, 76]. Sapp *et al*. [77] reported the feasibility of needle catheter ablation for targeting intramural substrates in refractory VT patients. The intramyocardial guidewire navigation technique offers a less invasive alternative, utilizing wire penetration into the myocardium to enable both mapping and ablation of intramural substrates [78].

Cauti *et al*. [79] analyzed the frequency characteristics of electrograms in VT activation maps. They reported that the lower frequency components were associated with a prolonged time to VT termination following high-frequency ablation, suggesting intramural circuit components [79]. The combination of high-density mapping and a detailed local electrogram analysis is expected to further elucidate the mechanisms of 3D VT circuits.

## 8. Limitations and Future Directions in Human VT Mapping

Despite major advances in VT mapping strategies, several important limitations remain unresolved. VT activation mapping continues to be restricted by the hemodynamic intolerance, often necessitating reliance on substrate-based approaches. Even with multielectrode catheters, accurate delineation of intramural substrates remains difficult, and electrogram interpretation is still subject to inter-operator variability. In addition, differences in mapping system algorithms and catheter design may contribute to variability in outcomes across centers, and further work toward standardization and validation of functional substrate criteria may help reduce such variability.

Looking forward, the integration of emerging technologies holds the potential to transform VT mapping. Artificial intelligence (AI) and machine learning may support the automated classification of electrograms and the prediction of critical isthmus regions, thereby reducing operator dependence and variability [80]. Real-time imaging, including photon-counting CT and advanced MRI, may complement functional mapping by providing high-resolution anatomical and scar characterization [81, 82]. Ultimately, the future of VT ablation might be characterized by hybrid approaches that combine functional mapping with AI-assisted analysis and multimodality imaging, applied according to individual cases, with the aim of improving accuracy, procedural efficiency, and long-term outcomes.

## 9. Conclusion

Our understanding of scar-related VT circuits has evolved through advances in mapping techniques, from early intraoperative mapping to contemporary high-density electroanatomical mapping. This evolution has not only deepened our mechanistic understanding of scar-related VT but has also transformed therapeutic strategies. Our aim is to eliminate life-threatening ventricular arrhythmias, reduce shock therapies delivered by implantable cardioverter-defibrillators, and improve patient outcomes. As technology continues to advance, efforts remain focused on refining mapping techniques and ablation strategies to enable a more effective and efficient treatment of scar-related VT.
